# Protective Effects of Cinnamaldehyde on the Oxidative Stress, Inflammatory Response, and Apoptosis in the Hepatocytes of *Salmonella* Gallinarum-Challenged Young Chicks

**DOI:** 10.1155/2022/2459212

**Published:** 2022-07-05

**Authors:** Lizi Yin, Sajjad Hussain, Ting Tang, Yuhong Gou, Changliang He, Xiaoxia Liang, Zhongqiong Yin, Gang Shu, Yuanfeng Zou, Hualin Fu, Xu Song, Huaqiao Tang, Funeng Xu, Ping Ouyang

**Affiliations:** College of Veterinary Medicine, Sichuan Agriculture University, Huimin Road 211, Wenjiang 611130, China

## Abstract

The development of novel therapeutics to treat multidrug-resistant pathogenic infections like *Salmonella gallinarum* is the need of the hour. *Salmonella* infection causes typhoid fever, jaundice, and *Salmonella* hepatitis resulting in severe liver injury. Natural compounds have been proved beneficial for the treatment of these bacterial infections. The beneficial roles of cinnamaldehyde due to its antibacterial, anti-inflammatory, and antioxidative properties have been determined by many researchers. However, alleviation of liver damage caused by *S. gallinarum* infection to young chicks by cinnamaldehyde remains largely unknown. Therefore, this study was performed to identify the effects of cinnamaldehyde on ameliorating liver damage in young chicks. Young chicks were intraperitoneally infected with *S. gallinarum* and treated with cinnamaldehyde orally. Liver and serum parameters were investigated by qRT-PCR, ELISA kits, biochemistry kits, flow cytometry, JC-1 dye experiment, and transcriptome analysis. We found that ROS, cytochrome c, mitochondrial membrane potential (*Ψ*m), caspase-3 activity, ATP production, hepatic CFU, ALT, and AST, which were initially increased by *Salmonella* infection, significantly (*P* < 0.05) decreased by cinnamaldehyde treatment at 1, 3, and 5 days postinfection (DPI). In addition, *S. gallinarum* infection significantly increased proinflammatory gene expression (*IL-1β*, *IL-6*, *IL-12*, *NF-κB*, *TNF-α*, and *MyD-88*) and decreased the expression of anti-inflammatory genes (*IL-8*, *IL-10*, and *iNOS*); however, cinnamaldehyde reverted these effects at 1, 3, and 5 DPI. Transcriptome analysis showed that *S. gallinarum* modulates certain genes of the AMPK-mTOR pathway for its survival and replication, and these pathway modulations were reversed by cinnamaldehyde treatment. We concluded that cinnamaldehyde ameliorates inflammation and apoptosis by suppressing NF-K*β*/caspase-3 pathway and reverts the metabolic changes caused by *S. gallinarum* infection via modulating the AMPK-mTOR pathway. Furthermore, cinnamaldehyde has antibacterial, anti-inflammatory, antioxidative, and antiapoptotic properties against *S. gallinarum-*challenged young chicks and can be a candidate novel drug to treat salmonellosis in poultry production.

## 1. Introduction

Family *Enterobacteriaceae* contains a genus named *Salmonella*, consisting of non-spore-forming facultative anaerobic bacilli [1]. This genus has two species, i.e., *Salmonella enterica* (*S. enterica*) and *S. bongori.* Five subspecies are placed under the category of *S. enterica* [2]. Humans and animals are mostly affected by *S. enterica* subspecies Enterica, which further contains typhoidal and nontyphoidal serovars [3]. More than 2600 serovars are present in *S. enterica* subspecies Enterica [4]. Annually, 93.8 million cases of nontyphoidal gastroenteritis have been reported worldwide, with 155,000 deaths and an average incidence of 1.14 episodes/100 person-years, making this disease a major public health concern [5]. 10–20 million cases of typhoid and about 100,000–200,000 deaths per annum have been reported recently [6]. Salmonellosis is not only a public health concern but also a major threat to the poultry industry as two nonmotile serovars of *Salmonella,* i.e., *S. enterica* serovar pullorum (causing pullorum disease) and *S. enterica* serovar gallinarum (causing fowl typhoid), are specific to birds and pose a serious impact on the business of poultry producers [7]. *Salmonella gallinarum* and *Salmonella* pullorum cause the systemic infection of poultry resulting in sepsis, independent of the age of birds [8].

*Salmonella*, a primary food-borne pathogen [9], makes its way to the intestine after being ingested with contaminated food. In the intestine, *Salmonella* causes intestinal dysbiosis by damaging the intestinal barrier function [9]. There is always the induction of a strong immune response involving cytokines and chemokines secretion in *S. typhimurium* infection, resulting in inflammatory damage [10]. Moreover, *Salmonella* also modulates the host metabolism for its benefit, evidenced by the upregulated glycolysis in the macrophages infected with *S. typhimurium* [11]. Complicated gut-liver interactions through the gut-liver axis act as a ladder in the liver injury during *Salmonella* infection, manifested by inflammatory cell infiltration, oxidative stress, hepatic apoptosis, and intense congestion [12].

Detoxification, nutrient metabolism, and immune response are some of the important physiological activities of the liver [13]. In humans, many factors like infection, drug exposure, and alcohol abuse contribute to the etiology of liver damage [14]. Liver injury due to *Salmonella* infection is an example of infection-associated liver damage [15]. Moreover, enteric or typhoid fever is a protracted febrile condition that is caused by *S. typhi* infection and characterized by abnormal levels of liver enzymes in the serum [16]. Kalia et al. also found increased serum alanine aminotransferase (ALT) and aspartate aminotransferase (AST) levels during *S. typhimurium* infection to mice [17]. A severe liver abnormality with jaundice is observed in the patients of typhoid fever, and this condition is termed *Salmonella* hepatitis [16]. In poultry, persistent infection of *S. gallinarum* and *S.* Pullorum has been observed, causing localization in the ovaries and resulting in vertical transmission by producing contaminated eggs [18]. Hepatomegaly, friable liver consistency and yellow-greenish color of the liver along with multifocal hepatic necrosis by the histopathology of birds infected with *S. gallinarum* have also been found [19].

Unsuccessful vaccination and antibiotic resistance against salmonellosis demand the development of novel therapeutics [20]. In this regard, bacteriostatic and anti-inflammatory activities of natural products have attracted researchers' attention [21]. Cinnamaldehyde is the main component of Chinese traditional medicine cassia twig and genus Cinnamon having several hundred species. Isolates from cinnamon plant are more than 89 while cinnamaldehyde is the major constituent [22, 23]. The beneficial roles of cinnamaldehyde due to its antibacterial, anti-inflammatory, and antioxidative properties have been determined. For example, immune response triggered by lipopolysaccharides in macrophages has been reduced by cinnamaldehyde treatment *in vitro* [24]. In the human dental pulp cells, cinnamaldehyde causes the alleviation of H_2_O_2_-induced oxidative stress by nuclear factor erythroid 2 (NF-E2) p45-related factor 2 (NRF2) and shows the heme oxygenase-1 (HO-1)-dependent antioxidant response [25]. Intestinal inflammatory response against *Cronobacter sakazakii* has been mitigated by cinnamaldehyde in the mice model, as it suppresses the *IL-1β*, *IL-6*, and *TNF-α* expression. It also causes the inhibition of the NF-*κ*B activation pathway and caspase-3 activity that leads to the reduction in enterocytic apoptosis [26]. A recent study indicated that the type-three secretion system (T3SS) of *Salmonella enterica* serovar Typhimurium had been inhibited by cinnamaldehyde as it affects the expression of key effector proteins in the mice model [21]. Despite these researches, evidence of mitigation of liver injury in the chicken models is largely unknown. Therefore, this study was performed to investigate the effects of cinnamaldehyde treatment on the morphological, physiological, pathological, biochemical, and metabolic changes caused by *S. gallinarum* infection to chicken hepatocytes at 1, 3, and 5 days postinfection (DPI). Furthermore, transcriptome analysis of 5 DPI hepatic tissue from control, challenge, and treatment groups was performed to get a deeper insight into the molecular mechanisms of cinnamaldehyde and gene expression changes at mRNA level in the *S. gallinarum*-challenged young chicks.

## 2. Materials and Methods

### 2.1. Animal Model, Sampling, and Ethical Issues

**Figure 1:**
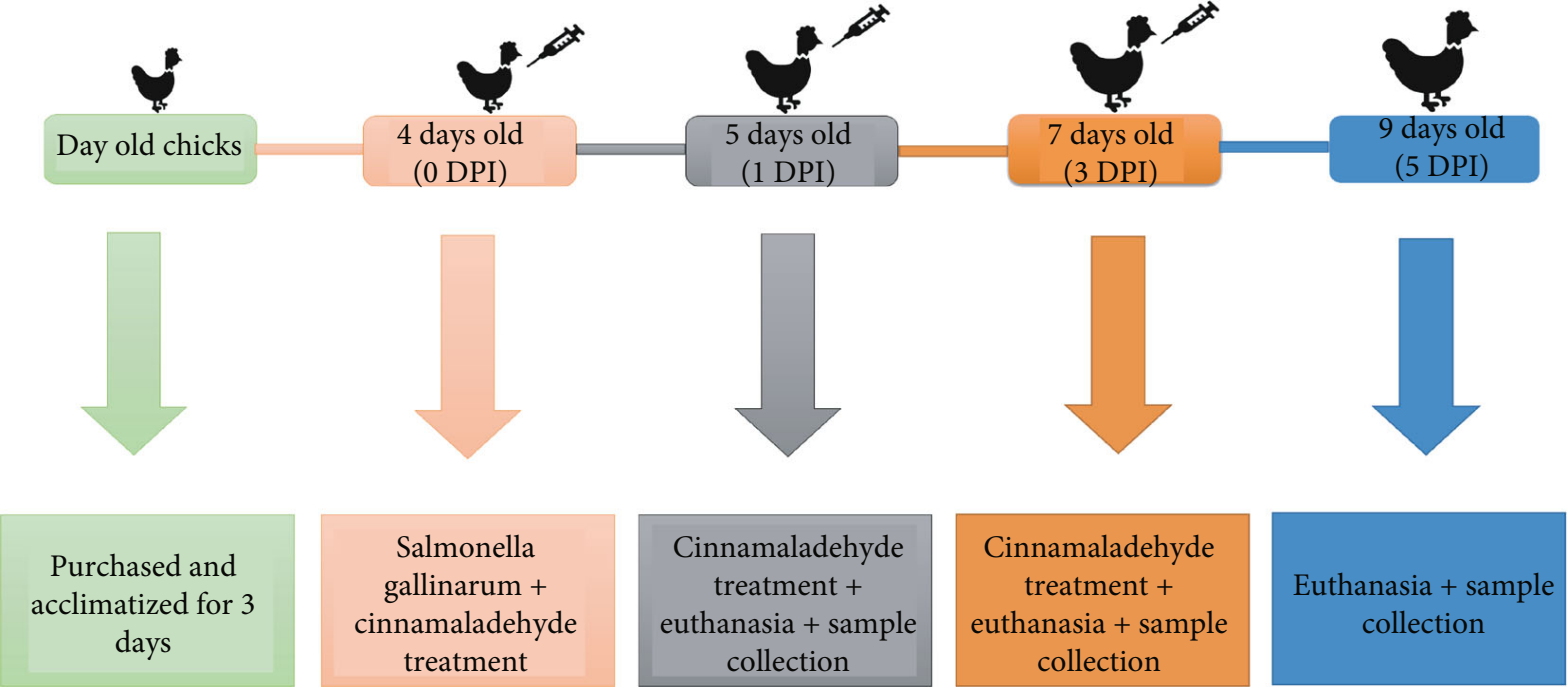
Experimental design. A total of 90 specific pathogen-free (SPF) day-old chicks were purchased and acclimatized for three days. The flock was divided into three groups, each with 30 chicks, i.e., control group (CON), challenge group (SG), and treatment group (SG+CA). At day 4 or 0 day postinfection (0 DPI), normal saline in the CON group, *S. gallinarum* in the SG group, and cinnamaldehyde in the SG+CA group were administered. Cinnamaldehyde in SG+CA was injected till 3 DPI. On day 5 (1 DPI), day 7 (3 DPI), and day 9 (5 DPI), blood samples were collected from 10 chicks (in each group) and euthanized on the respective days to collect liver samples.

A total of 90 specific pathogen-free (SPF) day-old chicks (Lohman broiler chicks, negative for *Salmonella*, bought from Chengdu Muxing poultry industry Co., Ltd.), weighing 50-56 g, were used in this study. After three days of acclimatization, the flock was divided into three groups, each with 30 chicks, i.e., control group (CON), challenge group (SG), i.e., infected with *S. gallinarum* (isolated from diseased chickens), and treatment group (SG+CA), i.e., infected with *S. gallinarum* and treated with cinnamaldehyde. 150 mg/kg body weight cinnamaldehyde in the SG+CA group (orally), 0.3 ml of 3 × 10^7^ CFU/ml *S. gallinarum* in SG group (intraperitoneally), and the same volume of normal saline (orally) in the CON group were injected. At 1, 3, and 5 DPI, blood samples were taken from 10 chicks (in each group). Chicks were euthanized (by cervical dislocation method) on the respective days. Livers were aseptically collected and cut into smaller pieces. Half of the samples were placed in an Eppendorf tube containing 4% formaldehyde, and the other half were immersed in liquid nitrogen for 10 minutes and stored in a -80° C freezer (see Figure 1). All experiments were conducted following the approved guidelines and experimental protocols by the Animal Care and Use Committee of Sichuan Agricultural University, China (permit number DKY-B2019603001).

### 2.2. Bacterial Colonization

The individual liver sample was weighed and subsequently transferred in sterilized mortar and pestle for maceration. The macerated liver contents were serially diluted (6-fold) in a sterilized 1% PBS solution. Later, tissue contents were plated on Salmonella-Shigella (SS) agar. Colonies typical for *Salmonella* were counted as Log_10_ CFU/g.

### 2.3. Estimation of ALT and AST

Blood samples were obtained and centrifuged to collect the serum. Serum ALT and AST levels were measured spectrophotometrically at an absorbance wavelength (505 nm) using commercial kits purchased from Nanjing Jiancheng Bioengineering Institute, Nanjing, China, and expressed as international units per litre (IU/l).

### 2.4. Quantitative Real-Time PCR

Total RNA was extracted following the manufacturer's instructions using a specialized kit (Aibimeng Biotechnology Co., Ltd. Zhenjiang, China). Precisely, 1 ml of TRIzol was added for 50 mg of liver tissue. Following lysis and centrifugation, the supernatant was transferred to an RNase-free centrifuge tube for subsequent purification steps. RNA was then eluted in 50 *μ*l of RNase-free water and quantified by the ratio of absorbance (260 nm/280 nm) using a Nanodrop spectrophotometer (Thermo Fisher Scientific, MA, USA). The eluted RNA was then stored at −70°C until use. Total RNA was transformed into cDNA using 5X All-In-One Master Mix (Applied Biological Materials, Vancouver, BC, Canada) following the manufacturer's instructions. After reverse transcription, the cDNA was collected and stored at -20°C. The qRT-PCR reaction was carried out by using abm® EvaGreen 2X qPCR Master Mix kit (Applied Biological Materials, Canada) on a CFX Connect™ Real-Time System (Bio-Rad Laboratories, Hercules, CA, USA) with specific primers (see Table 1) designed using the Primer Premier 6.0 software. Relative gene expression was calculated by the ratio of the target gene to reference gene (*β*-actin) expression. 2ˉ^*ΔΔ*Ct^ method [27] was used for the analysis of results.

### 2.5. Histopathological Examination

1 cm^2^ liver samples were taken from each group. 10% neutral-buffered formalin was used for tissue fixation. Tissue processing, trimming, and embedding in paraffin were performed subsequently. 5 *μ*m thick tissue sections were selected and stained with hematoxylin and eosin (H&E). Afterwards, the tissue sections were observed by a light microscope (Olympus, Tokyo, Japan).

### 2.6. Transmission Electron Microscopy (TEM) of Hepatic Tissue

At least 2 h fixation of liver samples was performed in 2.5% glutaraldehyde with a subsequent 2 h fixation in 1% osmium tetroxide. Later, a series of ethanol washes for gradient dehydration were applied on the samples and embedded in Epon 812 epoxy resin. Ultrathin sections (about 70 nm) were obtained by an ultramicrotome (EM UC6, Leica). Uranyl acetate and citrimalic acid lead were applied for double-staining. Samples were observed by TEM (Philips CM100) at 80 kV.

### 2.7. Caspase-3 Activity

The activity of caspase-3 in all the groups was measured using a commercial caspase-3 activity assay kit (Biyuntian Biotechnology Co., Ltd, Shanghai, China). 100 *μ*l of tissue lysing fluid (from the kit) was added to 3-10 mg of liver tissue in a sterilized Dounce homogenizer and homogenized. Homogenate was transferred to a 1.5 ml Eppendorf tube and placed on ice for 5 minutes. Centrifugation of the homogenate was done at 16000-20000 g for 10-15 minutes, and the supernatant was used for further experimentation following kit instructions.

### 2.8. Reactive Oxygen Species (ROS) Content in Chicken Hepatocytes

2,7-Dichloro-dihydro-fluorescein diacetate (DCFH-DA) was used to measure the ROS content in the hepatocytes following the kit instructions (Nanjing Jiancheng Bioengineering Institute, Nanjing, China). Approximately 1 mm^3^ liver tissue sample was taken and washed with PBS for subsequent digestion with trypsin enzyme at 37°C. The solution was screened using a nylon mesh, washed twice with PBS, and centrifuged to get a single-cell suspension. Later, DCFH-DA was added with dimethyl sulfoxide (DMSO) as solvent. The sample was incubated at 37° C for 1 hour in the dark, and fluorescence was observed at an optical excitation wavelength of 485 nm and an optical emission wavelength of 510 nm using a spectrophotometer (Thermo Fisher Scientific, Vantaa, Finland).

### 2.9. Estimation of Mitochondrial Membrane Potential (*Ψ*m) by JC-1 Experiment

Liver tissue was cut into 2-4 mm pieces with surgical blades. After gently blowing the dispersed cells with a pipette, remove the cells and debris with a filter and collect the cell suspension into a centrifuge tube for centrifugation and collection of the sediment. 10 mM of carbonyl cyanide 3-chlorophenylhydrazone (CCCP) was added to the positive group cells at the ratio of 1 : 1000, and the cells were treated for 20 minutes. 0.5 ml JC-1 staining solution was added, mixed, and incubated at 37° C for 20 minutes. Prepare proper amount of JC-1 dyeing buffer (1X) according to the ratio of JC-1 dyeing buffer (5X) : distilled water (1 : 4) and placed on ice. After incubation, centrifuge at 600 g for 3-4 minutes, precipitate cells, and discard the supernatant. Wash cells with JC-1 staining buffer (1X), add 1 ml of JC-1 staining buffer (1X) to resuspend cells, and centrifuge at 600 g for 3-4 minutes, precipitate cells, and discard the supernatant. JC-1 staining solution addition and incubation were done again at the same conditions and concentration. The cells were suspended with JC-1 staining buffer (1X) and analyzed by Beckman-Coulter Gallios flow cytometer (United States).

### 2.10. Cytochrome c Release Assay

Enzyme-linked immunosorbent assay (ELISA) was performed to measure cytochrome c concentration using a commercial ELISA kit (Nanjing Camilo Bioengineering Co., Ltd. Nanjing, China). 5-10 ml of tissue extraction reagent (from the kit) was added in 1 g of tissue and homogenized using sterilized mortar and pestle. Homogenate was centrifuged at 5000-10000 rpm for 10 minutes. The supernatant was collected to perform the next steps of the experiment following kit instructions.

### 2.11. Determination of ATP Concentration in Hepatocytes

Hepatocytic ATP concentration was assayed by spectrophotometry using a commercial kit purchased from Nanjing Jiancheng Bioengineering Institute, Nanjing, China. 30-50 mg of liver sample was taken and ground in 270 *μ*l double distilled water in a sterilized mortar and pestle. Eppendorf tubes were used for centrifugation at 3500 rpm for 10 minutes, and the supernatant was collected for further experiment. Absorbance was recorded at 636 nm, and ATP concentration was expressed as millimoles per gram protein (mmol/gram protein).

### 2.12. Transcriptome Analysis

Total RNA was extracted from the tissue using TRIzol® reagent, following the manufacturer's instructions (Invitrogen), and genomic DNA was removed by DNase I (TaKara). Then, RNA quality was determined by a 2100 Bioanalyzer (Agilent) and quantified using the ND-2000 (NanoDrop Technologies). Only high-quality RNA sample (OD 260/280 = 1.8 ~ 2.2, OD 260/230 ≥ 2.0, RIN ≥ 6.5, 28S : 18S ≥ 1.0, >1 *μ*g) was used to construct sequencing library. RNA-seq transcriptome library was prepared following the TruSeqTM RNA sample preparation kit from Illumina (San Diego, CA) using 1 *μ*g of total RNA. Shortly, mRNA was isolated according to the polyA selection method by oligo (dT) beads and then fragmented by fragmentation buffer firstly. Secondly, double-stranded cDNA was synthesized using a superscript double-stranded cDNA synthesis kit (Invitrogen, CA) with random hexamer primers (Illumina).

Then, the synthesized cDNA was subjected to end-repair, phosphorylation, and “A” base addition according to Illumina's library construction protocol. Libraries were size selected for cDNA target fragments of 300 bp on 2% Low Range Ultra Agarose followed by PCR, amplified using Phusion DNA polymerase (NEB) for 15 PCR cycles. After being quantified by TBS380, the paired-end RNA-seq sequencing library was sequenced with the Illumina HiSeq X Ten/NovaSeq 6000 sequencer (2 × 150 bp read length). The raw paired-end reads were trimmed, and quality was controlled by SeqPrep (https://github.com/jstjohn/SeqPrep) and Sickle (https://github.com/najoshi/sickle) with default parameters. Then, clean reads were separately aligned to reference genome with orientation mode using HISAT2 software. The mapped reads of each sample were assembled by StringTie in a reference-based approach [28]. To identify differentially expressed genes (DEGs) between two different samples, the expression level of each transcript was calculated according to the transcripts per million reads (TPM) method. RSEM [29] was used to quantify gene abundances. Essentially, differential expression analysis was performed using the DESeq2 [30]/DEGseq [31]/EdgeR [32] with *P* ≤ 0.05; DEGs with log2FC > 1 and *P* ≤ 0.05 (DESeq2 or EdgeR)/*P* ≤ 0.001 (DEGseq) were considered to be DEGs. In addition, functional-enrichment analyses including GO (gene ontology) and KEGG (Kyoto encyclopedia of genes and genomes) were performed to identify which DEGs were significantly enriched in GO terms and metabolic pathways at Bonferroni-corrected *P* ≤ 0.05 compared with the whole-transcriptome background. GO functional enrichment and KEGG pathway analysis were carried out by Goatools and KOBAS [33].

### 2.13. Statistical Analysis

All experiments were repeated at least thrice. Data are presented as the mean ± standard error of the mean (SEM). The statistical significance of differences was analyzed by unpaired two-tailed Student's *t*-test using GraphPad Prism 9 software. Differences with *P* < 0.05, 0.01, and 0.001 were considered statistically significant (∗), highly significant (∗∗), and extremely significant (∗∗∗), respectively.

## 3. Results

### 3.1. Cinnamaldehyde Decreases the Colony Counts of *S. gallinarum* in the Chicken Hepatocytes

**Figure 2:**
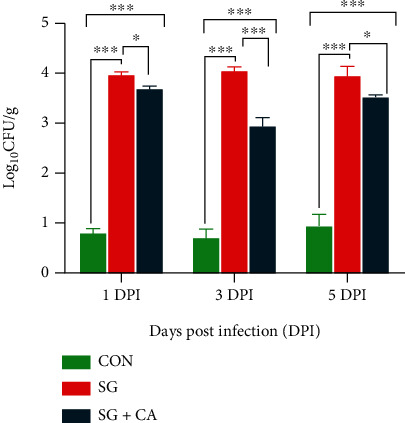
*S. gallinarum* colonization (CFU/g of hepatic tissue) in the chicken hepatocytes and attenuating effect of cinnamaldehyde at 1, 3, and 5 DPI. The control group is denoted by (CON), *S. gallinarum*-challenged group by (SG), and *S. gallinarum*-challenged, and the cinnamaldehyde treated group is represented by (SG+CA). Data are expressed as the mean ± SEM of three independent experiments. Significances were determined by a nonparametric test (^∗^*P* < 0.05,  ^∗∗^*P* < 0.01, and^∗∗∗^*P* < 0.001).

To determine the effect of cinnamaldehyde on the colonization of *S. gallinarum* in the chicken hepatic tissue, liver samples from all groups were taken and plated on SS agar to measure the colony counts at 1, 3, and 5 DPI. Data showed that there were significantly (*P* < 0.05) higher colony counts of *Salmonella* in the hepatic tissue from the SG group than that of the CON group at 1, 3, and 5 DPI (see Figure 2). However, the colony counts of *Salmonella* in the liver samples from the SG+CA group were significantly (*P* < 0.05) lower than that of the SG group at 1, 3, and 5 DPI. Hence, these data presented that cinnamaldehyde treatment decreased the *Salmonella* colonization in the chicken hepatocytes.

### 3.2. Cinnamaldehyde Decreases Serum AST and ALT Levels of *S. gallinarum*-Challenged Young Chicks

**Figure 3:**
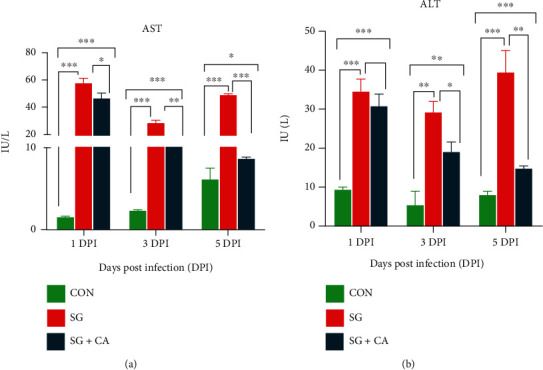
Effect of cinnamaldehyde on hepatic enzymes (AST and ALT) of chickens infected with *S. gallinarum* and treated with cinnamaldehyde. (a) Serum AST and (b) ALT levels in the chickens from control (CON), challenge (SG), and treatment (SG+CA) groups at 1, 3, and 5 DPI. Data are expressed as the mean ± SEM of three independent experiments. Significances were determined by nonparametric test (^∗^*P* < 0.05,  ^∗∗^*P* < 0.01, and^∗∗∗^*P* < 0.001).

A study demonstrated an increased serum AST and ALT concentrations after *S. typhimurium* infection in mice [17]. To find the effect of cinnamaldehyde treatment on the serum AST and ALT concentrations of *S. gallinarum*-challenged young chicks, serum AST and ALT levels were assayed. The concentrations of AST and ALT were significantly (*P* < 0.05) higher in the SG group at 1, 3, and 5 DPI than that of the CON group (see Figure 3). However, compared to the challenge (SG) group, the cinnamaldehyde treatment (SG+CA) group significantly (*P* < 0.05) decreased the serum AST and ALT concentrations at 1, 3, and 5 DPI. Hence, cinnamaldehyde treatment decreases serum AST and ALT levels of *S. gallinarum*-challenged young chicks.

### 3.3. Cinnamaldehyde Has Anti-inflammatory Properties in *S. gallinarum*-Challenged Chicken Hepatocytes

**Figure 4:**
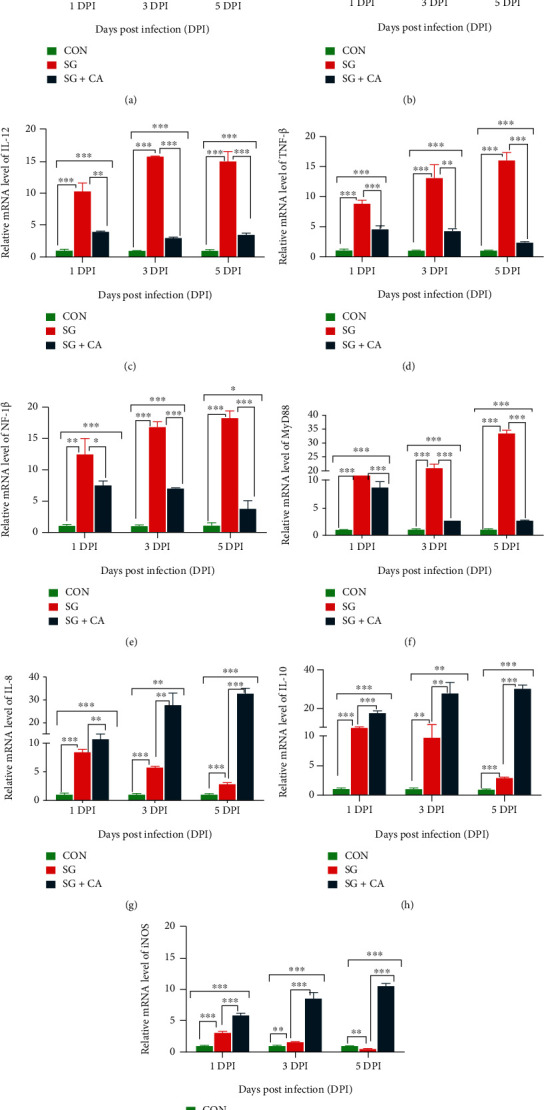
Effect of cinnamaldehyde on the expression levels of hepatic inflammation-related genes in chickens infected with *S. gallinarum*. (a–f) Relative mRNA level of pro-inflammatory genes (*IL-1β*, *IL-6*, *IL-12*, *TNF-α*, *NF-κB*, and *MYD-88*) and (g–i) anti-inflammatory genes (*IL-8*, *IL-10*, and *iNOS*) in the chicken hepatocytes of control (CON) group, challenge (SG) group and treatment (SG + CA) group at 1, 3, and 5 DPI. Data are expressed as the mean ± SEM of three independent experiments. Significances were determined by nonparametric test (^∗^*P* < 0.05,  ^∗∗^*P* < 0.01, and^∗∗∗^*P* < 0.001).

To assess the anti-inflammatory properties of cinnamaldehyde against *S. gallinarum* infection, relative mRNA expressions of certain proinflammatory and anti-inflammatory genes in hepatocytes of each group at 1, 3, and 5 DPI were determined using the qRT-PCR technique. Results indicated that the expression of proinflammatory genes (*IL-1β*, *IL-6*, *IL-12*, *NF-κB*, *TNF-α*, and *MyD-88*) were significantly (*P* < 0.05) higher in the SG group than that of SG+CA group at 1, 3, and 5 DPI (see Figure 4). However, the expression of anti-inflammatory genes (*IL-8*, *IL-10*, and *iNOS*) were significantly (*P* < 0.05) higher in the SG + CA group than that in the SG group. Hence, these data illustrated the anti-inflammatory properties of cinnamaldehyde in the hepatic tissue of *S. gallinarum*-challenged young chicks.

### 3.4. Cinnamaldehyde Protects against the Hepatic Morphological Injury Caused by *S. gallinarum*

**Figure 5:**
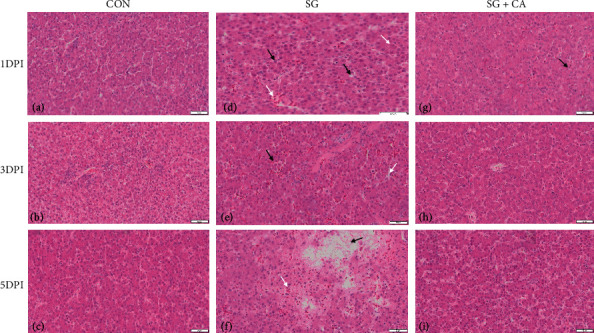
Histological findings in chicken hepatocytes challenged with *S. gallinarum* and treated with cinnamaldehyde in comparison with the control group at 1, 3, and 5 DPI. (a–c) represent the control (CON) groups, showing normal architecture of hepatic tissue (100X). (d) represents the challenge group (SG) at 1 DPI. Arrows indicate erythrocytes and congestion (white arrow) and lipid droplet formation (black arrow). (e) represents the challenge group (SG) at 3 DPI. Arrows indicate congestion (black arrow) and hepatic sinusoid (white arrow). (f) represents the challenge group (SG) at 5 DPI. Arrows indicate the necrotic foci (black arrow) and erythrocytes (white arrow). (g–i) show histology of hepatic tissue from the treatment (SG+CA) group at 1, 3, and 5 DPI. The black arrow in (g) indicates lipid droplet formation (100x).

**Figure 6:**
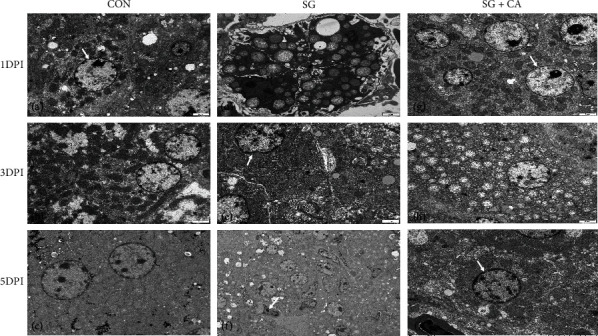
Effect of cinnamaldehyde on *S. gallinarum*-induced liver damage; evidence from transmission electron microscopy. (a–c) Liver of chicken from the control (CON) group showing the normal architecture of hepatocytes and smooth rounded nucleus with abundant normally distributed chromatin and prominent nucleolus, rough endoplasmic reticulum, and intact mitochondria. (d–f) Extensive liver damage in general and hepatocytic degeneration, in particular, have been found by intraperitoneal infection of *S. gallinarum* (SG group) at 1, 3, and 5 DPI by electron microscopy. However, (g–i) deleterious effects by intracellular activities of *Salmonella* were mitigated by cinnamaldehyde in the treatment (SG+CA) group, as evident by the figure showing relatively normal hepatocytic architecture compared to that of the challenge group (SG).

H&E staining (see Figure 5) and TEM (see Figure 6) of liver tissue indicated that intraperitoneal infection of *S. gallinarum* to young chicks resulted in hepatic colonization and disrupted normal hepatocytic architecture. Disruption of hepatic tissue was manifested by congestion, hemorrhages, and deformation of subcellular structures. However, cinnamaldehyde treatment reverted these morphological changes to a relatively normal state. Hence, cinnamaldehyde protects against the hepatic morphological injury caused by *S. gallinarum* infection.

### 3.5. Cinnamaldehyde Has Antiapoptotic Properties and Decreases ATP Concentration of *S. gallinarum*-Challenged Chicken Hepatocytes

**Figure 7:**
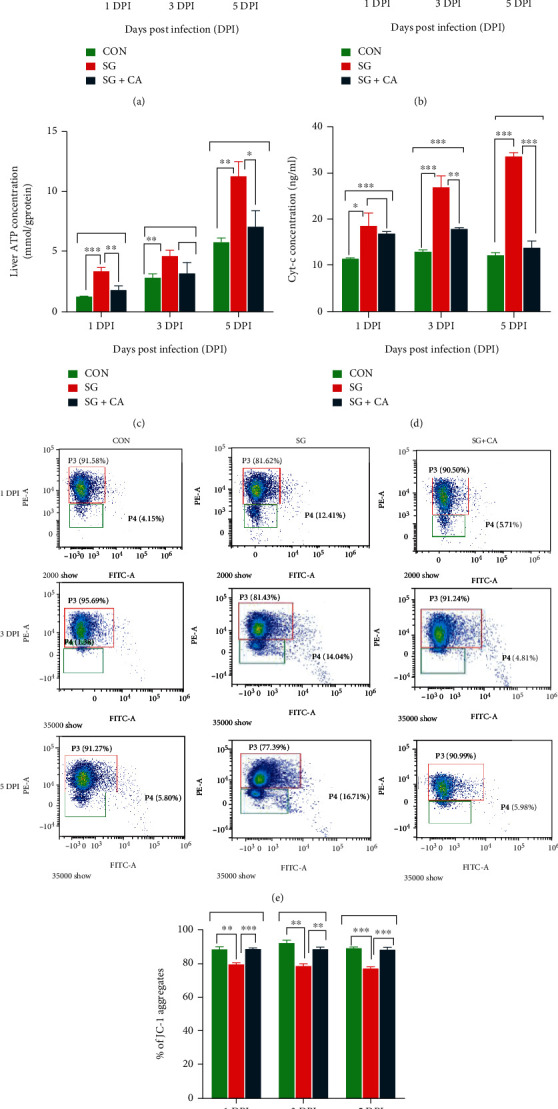
Antiapoptotic properties of cinnamaldehyde in the chicken hepatocytes challenged with *S. gallinarum*. (a) Hepatocytic reactive oxygen species (ROS) content, (b) activity of caspase-3, (c) ATP concentration, (d) cytochrome c concentration released in the cytosol, (e) flow cytometry results of JC-1 analysis to estimate mitochondrial membrane potential (*Ψ*m), and (f) percentage of JC-1 aggregates in the mitochondrial matrix of the chicken hepatocytes in the CON, SG, and SG+CA groups. Data are expressed as the mean ± SEM of three independent experiments. Significances were determined by nonparametric test (^∗^*P* < 0.05,  ^∗∗^*P* < 0.01, and^∗∗∗^*P* < 0.001). Note: when the mitochondrial membrane potential is high, JC-1 aggregates in the mitochondrial matrix and forms a polymer, which can produce red fluorescence and is received by the FITC channel. When the mitochondrial membrane potential is low, JC-1 could not aggregate in the mitochondrial matrix. At this time, JC-1 exists as a monomer and produce green fluorescence, which is received by the PE channel.

Host-pathogen interactions result in ROS production [34]. ROS production has an important role in regulating many cellular pathways, determining cell fate [35], oxidative stress, impairment of cellular metabolism, and apoptosis or necrosis [36]. To find the effect of cinnamaldehyde on the hepatocytic ROS production in the *S. gallinarum*-challenged young chicks, hepatocytic ROS content was measured in all groups at 1, 3, and 5 DPI. ROS content was significantly (*P* < 0.05) higher in the SG group than that of the CON group at 1, 3, and 5 DPI (see Figure 7(a)). However, we observed a significantly (*P* < 0.05) lower ROS production in the SG+CA group than that of the SG group at 1, 3, and 5 DPI. Hence, cinnamaldehyde treatment decreases ROS production in the *S. gallinarum*-challenged young chicks.

Increased ROS production causes a decrease in *Ψ*m [37]. To assess the mitochondrial membrane permeability transition due to *S. gallinarum* infection and treatment effect of cinnamaldehyde on it, the JC-1 experiment using flow cytometry technique was performed. We found a significant (*P* < 0.05) decrease in *Ψ*m in the SG group than that of the CON group at 1, 3, and 5 DPI. However, in the SG + CA group, *Ψ*m was significantly (*P* < 0.05) higher than that of the SG group at 1, 3, and 5 DPI (see Figures 7(e) and 7(f)). Therefore, cinnamaldehyde treatment protects the *S. gallinarum*-challenged chicken hepatocytes from the decrease in *Ψ*m.

During mitochondrial membrane permeabilization, cytochrome c is released from mitochondria to cytosol and activates caspases (like caspase-3) to initiate a cascade of events for apoptosis [38, 39]. To find the effect of cinnamaldehyde on the cytochrome c release from mitochondria of *S. gallinarum*-challenged chicken hepatocytes, ELISA was performed on the tissue sample from all groups at 1, 3, and 5 DPI. We found that in the SG group, the release of cytochrome c from mitochondria was significantly (*P* < 0.05) higher than that of the CON group at 1, 3, and 5 DPI (see Figure 7(d)). At the same time, cytochrome c release from mitochondria was significantly (*P* < 0.05) lower in the SG+CA group than that of the SG group at 1, 3, and 5 DPI. Hence, cinnamaldehyde treatment protects the chicken hepatocytes from cytochrome c release in the cytosol.

Enterocytic apoptosis is reduced by cinnamaldehyde treatment as it inhibits the caspase-3 activity [26]. Caspase-3 is the key enzyme for apoptosis [40]. As cytochrome c released from mitochondria activates caspases and apoptotic pathways, we performed the caspase-3 activity assay on the hepatocytes of *S. gallinarum*-challenged young chicks. We observed a significantly (*P* < 0.05) higher caspase-3 activity in the hepatocytes of the SG group than that of the CON group at 1, 3, and 5 DPI (see Figure 7(b)). However, a significantly (*P* < 0.05) lower caspase-3 activity in the SG+CA group than that of SG group was observed at 1, 3, and 5 DPI. Therefore, cinnamaldehyde treatment decreases the caspase-3 activity in the *S. gallinarum*-challenged chicken hepatocytes.

To find the effect of cinnamaldehyde on the energy content of *S. gallinarum*-challenged chicken hepatocytes, the concentration of ATP was determined in the CON, SG, and SG+CA groups at 1, 3, and 5 DPI. We observed that at 1, 3, and 5 DPI, chicken hepatocytes infected with *S. gallinarum* (SG group) exhibited significantly higher (*P* < 0.05) ATP concentration than that of the CON group (see Figure 7(c)). However, the SG+CA group showed a significantly (*P* < 0.05) lower hepatocytic ATP concentration than that of the SG group at 1, 3, and 5 DPI. Hence, cinnamaldehyde treatment decreases the ATP content of *S. gallinarum*-challenged chicken hepatocytes.

### 3.6. Cinnamaldehyde Reverses the Modulations of AMPK-mTOR Pathway Caused by *S. gallinarum*

**Figure 8:**
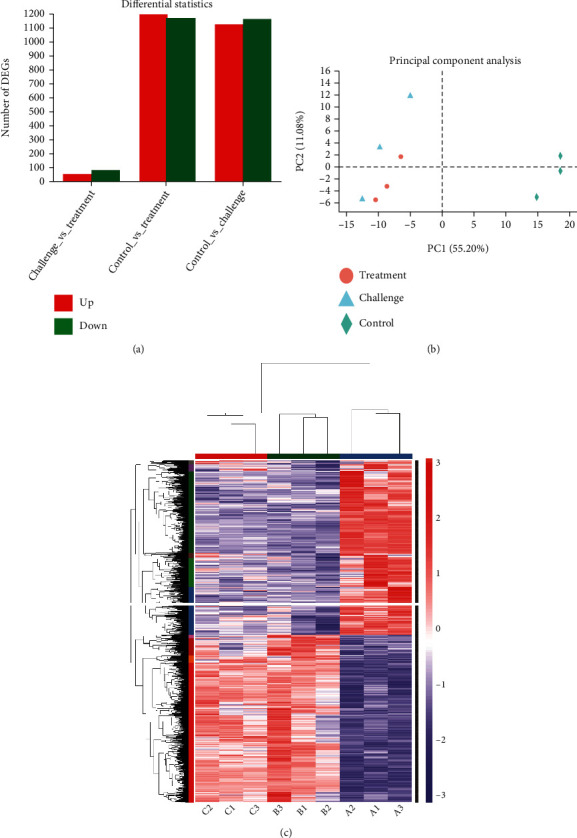
Number of differentially expressed genes (DEGs), principal component analysis (PCA), and heat map of the CON, SG, and SG+CA groups. (a) Graph showing statistics of DEGs between challenge_vs_treatment, control_ treatment, and control_vs_challenge where red and green colors show the number of up- and downregulated genes, respectively. (b) Principal component analysis (PCA) shows 55.20% and 11.08% variations between (PC1) and within (PC2) the samples, respectively. (c) Heat map showing the DEGs with log2FC > 1 at *P* < 0.05 in the CON, SG, and SG+CA groups.

Transcriptome analysis of chicken hepatocytes infected with *S. gallinarum* using RNA sequencing Illumina Platform (Hiseq xten/Nova seq 6000 Sequencing) was performed to identify the DEGs and understand the pathways involved in mechanism of action of cinnamaldehyde against *S. gallinarum* infection. Three individual repeats were selected from the CON, SG, and SG+CA groups at 5 DPI. High-quality data with an adequate sequencing depth was generated by high-throughput RNA sequencing. 2314 DEGs (see Figure 8(a)) were found in the control_vs_challenge group (1139 upregulated and 1175 downregulated), the control_vs_treatment group showed 2402 DEGs (1214 up-regulated and 1188 down-regulated), and the challenge_vs_treatment group exhibited 142 DEGs (56 upregulated and 86 downregulated). All raw transcriptome sequences were deposited in the National Center for Biotechnology Information (NCBI) sequence read archive (accession number: PRJNA784273). Principal component analysis (PCA) revealed 52.20% and 11.08% variance between the CON, SG, and SG+CA groups at PC1 and PC2, respectively (see Figure 8(b)). Moreover, the heat map showed a total of 2321 DEGs in the control_vs_treatment groups (see Figure 8(c)). Significant DEGs (fold change (FC) > 2 and a *P* < 0.05) were further subjected to GO and KEGG pathway functional classification.

Further, the GO classification of these significant DEGs between the above three groups was categorized into biological processes, cellular components, and molecular functions. We mainly focused on the challenge_vs_treatment group to find the mechanism of action of cinnamaldehyde in treating *Salmonella* infection to chicken.

**Figure 9:**
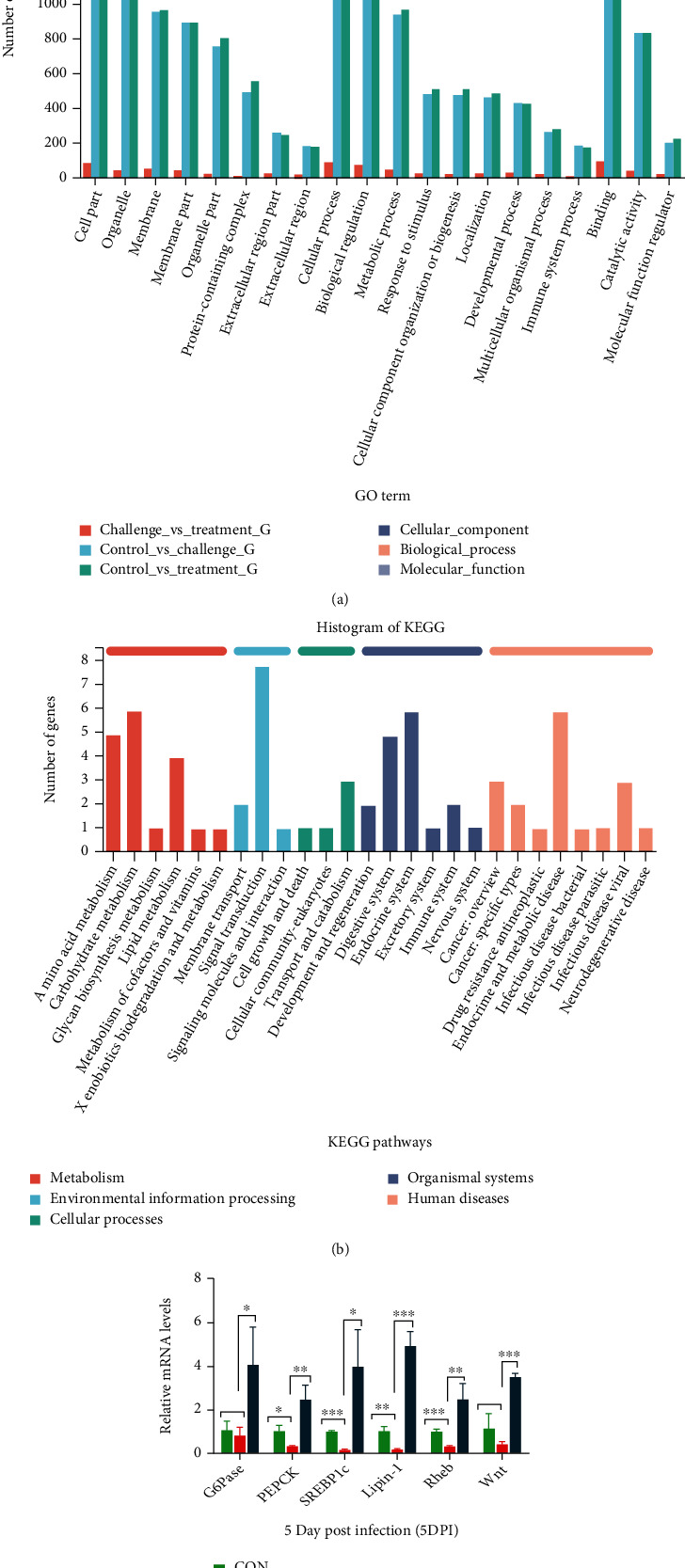
GO term enrichment analysis, histogram of KEGG, and validation of DEGs from transcriptome data by qRT-PCR. (a) Comparison of GO (gene ontology) term enrichment analysis. The *x*-axis shows the GO terms of biological processes, cellular components, and molecular function, and the *y*-axis indicates the number of genes. (b) represents the histogram of KEGG up-regulated genes in the challenge_vs_treatment group. (c) Validation of DEGs from transcriptome data by qRT-PCR indicating relative mRNA expression of *G6Pase*, *PEPCK*, *SREBP1*, *Lipin-1*, *Rheb*, and *Wnt* in the CON, SG, and SG+CA groups at 5 DPI. Data are expressed as the mean ± SEM of three independent experiments. Significances were determined by nonparametric test (^∗^*P* < 0.05,  ^∗∗^*P* < 0.01, and^∗∗∗^*P* < 0.001).

GO annotation analysis showed that the DEGs between the challenge group and the treatment groups were related to biological processes (such as metabolic processes and immunity), cellular components (such as organelle and membrane), and molecular functions (such as binding and catalytic activity). Differential genes for metabolic process and immunity were among the key upregulated DEGs in the biological processes (see Figure 9(a)).

KEGG is the principal public database of related pathways, often used in the research of RNA regulatory pathways and metabolic pathways [41]. The KEGG pathway annotation analysis is shown below (see Figure 9(b)). The pathways are classified into 7 categories: metabolism, environmental information processing, cellular processes, organismal systems, and human diseases. We selected the AMPK and mTOR signaling pathways to find the metabolism and immunity-related changes caused by infection and the effect of cinnamaldehyde treatment on the expression level of the genes involved in these pathways. We noticed that three genes, *ENSGALG00000027188* (*SREBP1*), *ENSGALG00000030034* (*G6Pase*), and *ENSGALG00000007636* (*PEPCK1*) working in AMPK pathway, were downregulated in the control_vs_challenge group, and their expressions were upregulated (in challenge_vs_treatment group) after the application of cinnamaldehyde. Moreover, two genes, *ENSGALG00000016456* (*Lipin-1*) and *ENSGALG00000041708* (*Wnt4*), which were downregulated in the control_vs_challenge group, were found upregulated in the challenge_vs_treatment group in the mTOR pathway. Upregulated genes by cinnamaldehyde in AMPK pathway, i.e., *SREBP1*, *G6Pase*, and *PEPCK1*, were involved in gluconeogenesis and fatty acid biosynthesis. At the same time, upregulated genes by medicine in the mTOR pathway, i.e., *Lipin-1* and *Wnt4*, were involved in lipid and protein biosynthesis, respectively. Hence, cinnamaldehyde reverses the modulations of the AMPK-mTOR pathway caused by *S. gallinarum*. To validate the expression of DEGs obtained in RNA-seq data, we selected five genes among up- and downregulated DEGs and determined their relative expression by qRT-PCR. Consequently, qRT-PCR results were consistent with RNA-seq data (see Figure 9(c)).

## 4. Discussion

Components from traditional Chinese medicine (e.g., costunolide and cinnamaldehyde) have been proved effective in mitigating the liver damage [42]. In this study, we found that *S. gallinarum* infection to chicken resulted in many hepatic physiological and pathological changes which were abrogated by cinnamaldehyde treatment. We observed that mechanism of action of cinnamaldehyde in treating hepatic damage caused by *Salmonella gallinarum* infection to chicken hepatocytes was similar to Mao et al.'s study who demonstrated that costunolide is effective in treating acute liver injury by promoting antioxidative defense system, suppressing inflammatory response, and preventing hepatocytic apoptosis [43]. Lesions caused by infection and the protective efficiency of cinnamaldehyde were manifested by H&E staining and TEM. Previous research shows that intraperitoneal infection of *Salmonella* resulted in the heterophils aggregates in the parenchyma of chicken hepatocytes [44]. Similarly, oral infection of 1-week-old Rhode Red Island chickens with *S. typhimurium* resulted in bacterial colonization in the livers [45]. Our findings were consistent with these studies as we found bacterial colonization in liver of chickens infected with *S. gallinarum*. Results of our study showing the efficiency of cinnamaldehyde in reducing bacterial counts were also in accordance with Johny et al. [46], who found that trans-cinnamaldehyde reduces the plate count of *Salmonella* strains in cinnamaldehyde-treated water.

Destruction of hepatic tissue leads to hepatocytic damage and an increase in the permeability of the hepatocytic plasma membrane, which is manifested by ALT and AST release into the blood from hepatocytes [47]. The level of these enzymes is proportional to the number of damaged hepatocytes [48]. Hence, hepatic injury can be predicted by measuring the level of these enzymes in the blood. Our results showed an increase in the ALT and AST levels after *Salmonella* infection to the chicken. At the same time, the treatment group exhibited decreased ALT and AST levels than that of the challenge group. These findings were consistent with Wang et al.'s [49] work of cinnamaldehyde effect on *S. typhimurium-*challenged mouse hepatocytes.

Hepatic damage in animals results in the biosynthesis and secretion of proinflammatory cytokines and chemokines [14]. In our experiment, the expression of proinflammatory genes of the hepatocytes was analyzed to find their implication and beneficial effects of cinnamaldehyde on them. Wang et al. [49] also found the effects of cinnamaldehyde on the expression of proinflammatory genes of the hepatocytes in a mouse model. Our findings were consistent with their study as *S. gallinarum* significantly increased the expression of proinflammatory genes (*IL-1β*, *IL-6*, *IL-12*, *NF-κB*, *TNF-α*, and *MyD-88*). However, cinnamaldehyde treatment, as expected, significantly decreased proinflammatory genes expression and increased the expression of anti-inflammatory genes (*IL-8*, *IL-10*, and *iNOS*), than that of the challenge group. Furthermore, we speculated that *S. gallinarum* infection-induced inflammatory damage could be suppressed by cinnamaldehyde in MyD-88 dependent signaling. However, there is a need for further investigation in this regard.

The control group showed a complete and normal architecture of hepatocytes in light and electron microscope, as evident from Figures 5 and 6, respectively; hence, it was used to compare the challenge and treatment groups. The most common histological lesions observed included necrosis, degeneration, hemorrhages, and infiltration of inflammatory cells. We found less tissue damage and relatively normal hepatocytic architecture in the treatment group than that of the challenge group, indicating that cinnamaldehyde has a protective effect on the hepatic tissue against *S. gallinarum* infection, and these findings are consistent with another study in which a mouse was used as a model animal [49].

ROS are produced by host-pathogen interactions [34]. *S. typhimurium* causes an increase in the ROS production in the Henle-407 cells time-dependently [50]. Clearing bacteria through ROS production by phagocytes makes ROS an important component of the innate immune response [51]. We found increased ROS production in the cells infected with *S. gallinarum* at 1, 3, and 5 DPI. At the same time, cinnamaldehyde significantly decreased ROS production in the infected group of chicks. These findings were consistent with Yang et al. [52], who found that cinnamaldehyde, *in vitro*, inhibited ROS production in high-glucose induced dorsal root ganglion (DRG) neurons dose-dependently. Hence, we found that cinnamaldehyde treatment has anti-oxidative properties in the hepatocytes of the *S. gallinarum*-challenged young chicks.

An increased ROS production results in a decreased *Ψ*m [37]. A decrease in the *Ψ*m has been observed by the *S. typhi* plasmid infection to macrophages that result in apoptosis [53]. Lv et al. [54] found the antiapoptotic properties of cinnamaldehyde as it protected the PC12 cells from a glutamate-induced decrease in *Ψ*m. Our results were consistent with these findings as *S. gallinarum-*infected hepatocytes showed decreased *Ψ*m. In contrast, cinnamaldehyde protected this decrease in *Ψ*m, exhibiting its antiapoptotic role during *S. gallinarum* infection to chickens.

Research shows that in *S. typhimurium*-infected macrophages (*in vitro*), cytochrome c is released from mitochondria [55]. The released cytochrome c activates a cascade of events involved in apoptosis [38]. Consistent with these studies, we found higher cytochrome c in the challenge group than that of the control group. At the same time, a significant decrease in cytochrome c in the treatment group than that of the challenge group was observed. These findings are consistent with the study of Lv et al., who found a dose-dependent decrease in the cytochrome c concentration in glutamate-induced PC12 cells treated with cinnamaldehyde [54].

**Figure 10:**
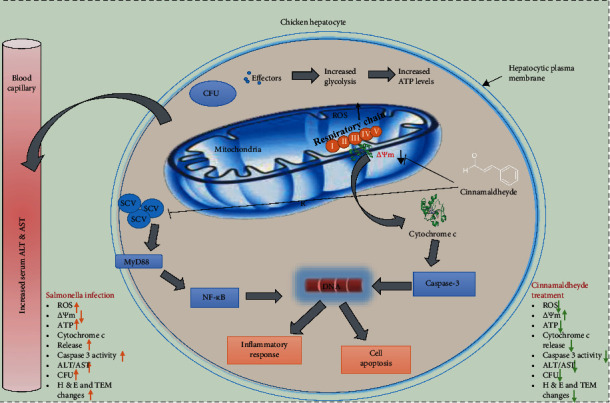
Summary of events of *S. gallinarum* infection to chicken hepatocytes and protective effects of cinnamaldehyde. *S. gallinarum* infection to chicken hepatocytes disrupts the electron transport chain resulting in high ROS production, decrease in *Ψ*m, and cytochrome c release from mitochondria. Cytochrome c triggers caspase-3 activity, which eventually causes apoptosis. Bacterial effectors result in increased glycolysis and ATP production. At the same time, increased hepatic CFU, MyD88/NF-*κ*B dependent inflammatory response, serum AST, and ALT levels were also found in *S. gallinarum*-infected chicken hepatocytes. In contrast, cinnamaldehyde treatment resulted in decreased ROS production and relatively normal mitochondrial membrane potential, and cytochrome c release was significantly lower than that of the infected group. Moreover, caspase-3 activity and extent of apoptosis were also significantly lower than that of the challenge group. Similarly, cinnamaldehyde protects the host from metabolic changes, indicated by significantly lower ATP concentration in the treatment group than that of the challenge group. Lower ALT and AST levels, inflammatory response, H&E changes, TEM changes, and hepatic CFU were also observed in the treatment group.

Caspase-3 is the key enzyme for apoptosis [40]. After *Salmonella* infection, increased caspase-3 activity has been detected in monocyte-derived macrophages [55]. *S. typhimurium* infection to mice results in the caspase-3 dependent phagocytic apoptosis [56]. In our experiment, we found a significant increase in caspase-3 activity during *S. gallinarum* infection, while cinnamaldehyde treatment decreased caspase-3 activity significantly at 1, 3, and 5 DPI. These findings were similar to Yang et al.'s work which showed that cinnamaldehyde significantly suppressed overexpression of cleaved caspase-3 in high-glucose- (HG-) induced dorsal root ganglion (DRG) neurons *in vitro* [52]. As the decrease in *Ψ*m and increase in ROS production, cytochrome c release, and caspase activity are involved in apoptosis, and cinnamaldehyde treatment protects these phenomena (see Figure 10), we concluded that cinnamaldehyde has antiapoptotic properties against *S. gallinarum*-challenged chicken hepatocytes.

Upregulated glycolysis has been observed in the macrophages infected with *S. typhimurium* [11]. Similarly, metabolic activities in the gut are induced and promoted by *Salmonella* for its growth and survival [57]. Cinnamaldehyde treatment in mice causes gluconeogenesis, glucose storage, and inhibition of glycolysis [58]. Like *S. typhimurium*, *Chlamydia* species are also obligated intracellular pathogens that replicate only within the infected cells. *Chlamydia* infection results in increased ATP production, with a peak level occurring midway through the infection cycle where most metabolically active bacteria are proliferating [59]. Consistent with these studies, we found an increased ATP concentration in the hepatocytes infected with *Salmonella*, while a decrease in hepatocytic ATP content was found in the group treated with cinnamaldehyde. Hence, we assumed that cinnamaldehyde treatment protects chicken hepatocytes from going into a higher metabolic state for increased ATP production compelled by the *S. gallinarum* infection.

**Figure 11:**
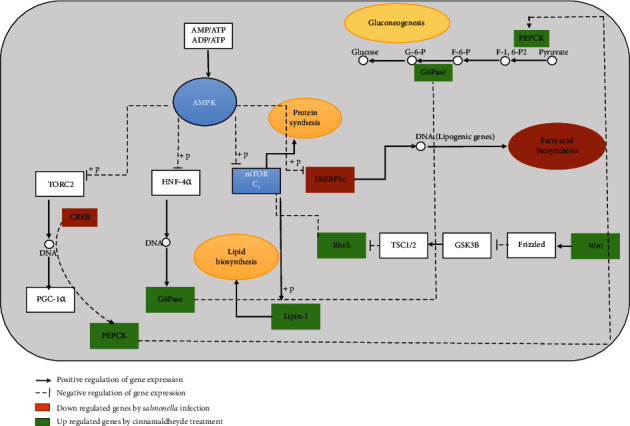
Modulation of AMPK-mTOR pathway by *S. gallinarum* infection to chicken hepatocytes and reversing effect of cinnamaldehyde; an evidence from transcriptome analysis. *S. gallinarum* infection results in activation of the AMPK-mTOR pathway, eventually causing less glucose, fatty acid, protein, and lipid biosynthesis. At the same time, cinnamaldehyde treatment inhibits these metabolic changes and results in the modulation of the genes involved in more glucose, fatty acid, protein, and lipid biosynthesis. It is important to note that genes of AMPK-mTOR, which were upregulated in the treatment group, were actually downregulated in the challenge group and vice versa.

Immunometabolism (interface of the immune system and metabolism) is an emerging field of research [60]. Within poultry production, considered immunometabolic consequences are not valued, and the sole focus is on maximizing growth, which decreases immune potential. Distinct immune and metabolic pathways are changed between 2-4 days postinfection, resulting in the alterations of the local metabolic environment. AMPK and mTOR pathways are an important link between the extracellular environment and intracellular metabolism during *Salmonella* infection to young chickens [61]. As the first week is very crucial for the growth of broiler chickens because of higher metabolism and relatively weaker immune system than that of later stages [62], we selected the modulations caused by cinnamaldehyde in the AMPK and mTOR pathways at 5 DPI. DEGs obtained by the transcriptome analysis showed that genes of the AMPK-mTOR pathway, which were upregulated in the treatment group, were downregulated in the challenge group and vice versa. *S. gallinarum* infection activated the AMPK-mTOR pathway, eventually resulting in less gluconeogenesis, fatty acid, protein, and lipid biosynthesis. These results are consistent with Jiang et al. [11], who showed that decreased glucose levels in macrophages after *S. typhimurium* infection led to upregulated bacterial uptake of 2- and 3-phosphoglycerate and phosphoenolpyruvate (carbon source) while on the other hand, cinnamaldehyde treatment caused more gluconeogenesis, fatty acid, protein, and lipid biosynthesis (bring to a normal level). Gluconeogenesis, glucose storage, and inhibition of glycolysis are also observed by cinnamaldehyde treatment in mice [58]. In our study, downregulation of *CREB*, *PEPCK*, and *G6Pase* genes of the AMPK pathway in *Salmonella* infection eventually resulted in less glucose production (see Figure 11). However, downregulation of mTOR pathway genes, i.e., *Lipin-1*, *Rheb*, and *SREBP1c* genes in *S. gallinarum* infection, reflect less lipid, protein, and fatty acid biosynthesis, respectively. In contrast to this, cinnamaldehyde treatment upregulated *PEPCK* and *G6Pase* genes (AMPK pathway), which promote gluconeogenesis. Upregulation of *Lipin-1* and *Rheb* genes (mTOR pathway) in the cinnamaldehyde treatment group indicates the promoted lipid biogenesis and protein synthesis, respectively. Hence, it is speculated that cinnamaldehyde reverses the AMPK-mTOR pathway changes caused by *S. gallinarum* infection.

## 5. Conclusion

Salmonellosis is a major concern to public health and poultry producers. Unsuccessful vaccination and antibiotic resistance against *Salmonella* infections demand researchers to develop novel therapeutics. Results of the current study revealed the protective effects of cinnamaldehyde on oxidative stress, inflammatory response, and apoptosis on the hepatocytes of *Salmonella gallinarum*-challenged young chicks. *S. gallinarum* induces inflammation and apoptosis via NF-K*β*/caspase-3-dependent pathway in the hepatocytes of young chicks. In addition, *S. gallinarum* infection may also result in metabolic changes via modulating the AMPK-mTOR pathway. However, cinnamaldehyde treatment ameliorated inflammation and apoptosis caused by *Salmonella* infection by suppressing NF-K*β*/caspase-3 pathway. Transcriptome analysis revealed that cinnamaldehyde treatment reverted the metabolic changes caused by *S. gallinarum* infection via the AMPK-mTOR pathway. Nonetheless, we only focused on investigating the effect of cinnamaldehyde on the hepatic tissue of *S. gallinarum*-challenged young chicks in the early days of life as this period is crucial for the growth of poultry birds because of higher metabolism and lower immunity than that of the later stages. In the future, long-term experimental studies and gene knockout systems can be used to validate the genes involved in the mechanism of action of cinnamaldehyde in the mitigation of liver injury caused by *Salmonella gallinarum* infection to chicken. We concluded that cinnamaldehyde has antibacterial, anti-inflammatory, antioxidative, and antiapoptotic properties against *S. gallinarum* infection to chicken hepatocytes and it can be a candidate drug for the treatment of *Salmonella* infections in poultry production.

## Figures and Tables

**Table 1:** All primers for quantitative real-time PCR used in the study.

Target	Sequence (5′→3′)	Gene bank accession number
TNF-*α*-F	TGTCGGTCAGCCGCTTCTC	MF000729.1
TNF-*α*-R	TGGTCGCCTCCAACTCGTC	
IL-1*β*-F	GGTCAACATCGCCACCTACA	XM_015297469.2
IL-1*β*-R	CATACGAGATGGAAACCAGCAA	
IL-6-F	AAATCCCTCCTCGCCAATCT	HM179640.1
IL-6-R	CCCTCACGGTCTTCTCCATAAA	
IL-8-F	CCATCTTCCACCTTTCACA	HM179639.1
IL-8-R	ATCCCACAGCACTGACCAT	
IL-10-F	CGGGAGCTGAGGGTGAA	NM_001004414.2
IL-10-R	GTGAAGAAGCGGTGACAGC	
IL-12-F	TGAGAGTCCAAGTGTGAAGTTC	DQ202328.1
IL-12-R	TCTTCGGCAAATGGACAGTAG	
NF-*κ*B-F	TCAACGCAGGACCTAAAGACAT	AF000241.1
NF-*κ*B-R	GCAGATAGCCAAGTTCAGGATG	
MyD88-F	CTGGCATCTTCTGAGTAGT	NM_001030962.5
MyD88-R	TTCCTTATAGTTCTGGCTTCT	
iNOS-F	CCTGGAGGTCCTGGAAGAGT	EF178279.1
iNOS-R	CCTGGGTTTCAGAAGTGGC	
Wnt1-F	GATCGTTAACAGGGGGTGCC	XM_015273182.3
Wnt1-R	GGTAATCGCAGGTGCACGAT	
G6Pase-F	CACCCTGCAGCCTGTTTTTC	XM_004948631.3
G6Pase-R	CCACAGGTTCGTGTTTGCTG	
Rheb-F	ATCAGACCGCCGTTGATGTT	XM_015281239.3
Rheb-R	ACTTCCCTTGTGAAGCTGCC	
Lipin 1-F	AGCCTGGTACTGAAACATCT	XM_040666999.1
Lipin 1-R	CTTGCCTTTGTAGCCTGTG	
PEPCK-F	GTTACCTGGAACATTGGCTGTC	J05419.1
PEPCK-R	CAGGTCCAAATCCCCTTCTT	
SREBP1-F	CTACCGCTCATCCATCAACG	AY029224.1
SREBP1-R	CTGCTTCAGCTTCTGGTTGC	
*β*-Actin-F	GGTATGGGCCAGAAAGAC	NM_205518.2
*β*-Actin-R	CTCCTCACGGGCTACTCT	

## Data Availability

Sequencing data are available on NCBI (PRJNA784273). The data used to support the findings of this study are available from the corresponding author upon request.

## References

[1] Chen S. H., Parker C. H., Croley T. R., McFarland M. A. (2019). Identification of Salmonella taxon-specific peptide markers to the serovar level by mass spectrometry. *Analytical Chemistry*.

[2] Desvaux M., Hébraud M., Henderson I. R., Pallen M. J. (2006). Type III secretion: what's in a name?. *Trends in Microbiology*.

[3] Engel A. C., Herbst F., Kerres A., Galle J. N., Hegemann J. H. (2016). The type III secretion system-related CPn0809 from chlamydia pneumoniae. *PLoS One*.

[4] Saleh S., Van Puyvelde S., Staes A. (2019). *Salmonella* Typhi, Paratyphi A, Enteritidis and typhimurium core proteomes reveal differentially expressed proteins linked to the cell surface and pathogenicity. *PLoS Neglected Tropical Diseases*.

[5] Majowicz S. E., Majowicz S. E., Musto J. (2010). The Global Burden of Nontyphoidal *Salmonella* Gastroenteritis. *Clinical Infectious Diseases*.

[6] Antillón M., Warren J. L., Crawford F. W. (2017). The burden of typhoid fever in low- and middle-income countries: a meta-regression approach. *PLoS Neglected Tropical Diseases*.

[7] Mahmud M. S., Bari M. L., Hossain M. A. (2011). Prevalence of*Salmonella*Serovars and antimicrobial resistance profiles in poultry of Savar Area, Bangladesh. *Foodborne Pathogens and Disease*.

[8] Cardoso W. M., Teixeira R. S. C., Albuquerque Á. H. (2013). Salmonella gallinarum virulence in experimentally-infected Japanese quails (Coturnix japonica). *Brazilian Journal of Poultry Science*.

[9] Dong N., Li X., Xue C. (2019). Astragalus polysaccharides attenuated inflammation and balanced the gut microflora in mice challenged with Salmonella typhimurium. *International Immunopharmacology*.

[10] Eckmann L., Kagnoff M. F. (2001). Cytokines in host defense against *Salmonella*. *Microbes and Infection*.

[11] Jiang L., Wang P., Song X. (2021). *Salmonella* typhimurium reprograms macrophage metabolism via T3SS effector SopE2 to promote intracellular replication and virulence. *Nature Communications*.

[12] Wei S., Huang J., Liu Z. (2019). Differential immune responses of C57BL/6 mice to infection by Salmonella enterica serovar Typhimurium strain SL1344, CVCC541 and CMCC50115. *Virulence*.

[13] Nguyen T. T., Almon R. R., DuBois D. C., Sukumaran S., Jusko W. J., Androulakis L. P. (2014). Tissue-specific gene expression and regulation in liver and muscle following chronic corticosteroid administration. *Gene Regulation and Systems Biology*.

[14] Zhang Y., Jia H., Jin Y. (2020). Glycine attenuates LPS-induced apoptosis and inflammatory cell infiltration in mouse liver. *The Journal of Nutrition*.

[15] Song F., Liu J., Zhao W. (2020). Synergistic effect of eugenol and probiotic Lactobacillus plantarum Zs2058 against salmonella infection in C57bl/6 mice. *Nutrients*.

[16] González-Quintela A., Campos J., Alende R. (2004). Abnormalities in liver enzyme levels during Salmonella enteritidis enterocolitis. *Revista Espanola de Enfermedades Digestivas*.

[17] Kalia P., Kumar N. R., Harjai K. (2015). The therapeutic potential of propolis against damage caused by Salmonella typhimurium in mice liver: a biochemical and histological study. *Archives of Biological Sciences*.

[18] Freitas Neto O. C., Arroyave W., Alessi A. C., Fagliari J. J., Berchieri A. (2007). Infection of commercial laying hens with Salmonella gallinarum: clinical, anatomopathological and haematological studies. *Brazilian Journal of Poultry Science*.

[19] Berchieri A., Murphy C. K., Marston K., Barrow P. A. (2001). Observations on the persistence and vertical transmission ofSalmonella entericaserovars pullorum and gallinarum in chickens: effect of bacterial and host genetic background. *Avian Pathology*.

[20] Hussain S., Ouyang P., Zhu Y. (2021). Type 3 secretion system 1 of salmonella typhimurium and its inhibitors: a novel strategy to combat salmonellosis. *Environmental Science and Pollution Research*.

[21] Liu Y., Zhang Y., Zhou Y. (2019). Cinnamaldehyde inhibits type three secretion system in *Salmonella* enterica serovar Typhimurium by affecting the expression of key effector proteins. *Veterinary Microbiology*.

[22] Jayaprakasha G., Rao L. J. M. (2011). Chemistry, biogenesis, and biological activities of Cinnamomum zeylanicum. *Critical Reviews in Food Science and Nutrition*.

[23] Rao P. V., Gan S. H. (2014). Cinnamon: a multifaceted medicinal plant. *Evidence-based Complementary and Alternative Medicine*.

[24] Kim M. E., Na J. Y., Lee J. S. (2018). Anti-inflammatory effects of trans-cinnamaldehyde on lipopolysaccharide-stimulated macrophage activation via MAPKs pathway regulation. *Immunopharmacology & Immunotoxicology*.

[25] Kim N. Y., Ahn S. G., Kim S. A. (2017). Cinnamaldehyde protects human dental pulp cells against oxidative stress through the Nrf2/HO-1-dependent antioxidant response. *European Journal of Pharmacology*.

[26] Yang G., Jin T., Yin S. (2019). trans-Cinnamaldehyde mitigated intestinal inflammation induced by Cronobacter sakazakii in newborn mice. *Food & Function*.

[27] Livak K. J., Schmittgen T. D. (2001). Analysis of relative gene expression data using real-time quantitative PCR and the 2^−*ΔΔ* _C_^_T_ method. *Methods*.

[28] Kim D., Langmead B., Salzberg S. L. (2015). HISAT: a fast spliced aligner with low memory requirements. *Nature Methods*.

[29] Trapnell C., Williams B. A., Pertea G. (2010). Transcript assembly and quantification by RNA-Seq reveals unannotated transcripts and isoform switching during cell differentiation. *Nature Biotechnology*.

[30] Pertea M., Pertea G. M., Antonescu C. M., Chang T. C., Mendell J. T., Salzberg S. L. (2015). StringTie enables improved reconstruction of a transcriptome from RNA-seq reads. *Nature Biotechnology*.

[31] Buchfink B., Xie C., Huson D. H. (2015). Fast and sensitive protein alignment using DIAMOND. *Nature Methods*.

[32] Finn R. D., Clements J., Eddy S. R. (2011). HMMER web server: interactive sequence similarity searching. *Nucleic Acids Research*.

[33] Liao Y., Smyth G. K., Shi W. (2014). featureCounts: an efficient general purpose program for assigning sequence reads to genomic features. *Bioinformatics*.

[34] Zeng L., Zhou J., Li B., Xing D. (2015). A high-sensitivity optical device for the early monitoring of plant pathogen attack via the in vivo detection of ROS bursts. *Frontiers in Plant Science*.

[35] Pierre A. S., Minville-Walz M., Fèvre C. (2013). Trans-10, cis-12 conjugated linoleic acid induced cell death in human colon cancer cells through reactive oxygen species-mediated ER stress. *Biochimica et Biophysica Acta*.

[36] Onyango I. G. (2008). Mitochondrial dysfunction and oxidative stress in Parkinson’s disease. *Neurochemical Research*.

[37] Chen H., Yoshioka H., Kim G. S. (2011). Oxidative stress in ischemic brain damage: mechanisms of cell death and potential molecular targets for neuroprotection. *Antioxidants & Redox Signaling*.

[38] Brentnall M., Rodriguez-Menocal L., de Guevara R. L., Cepero E., Boise L. H. (2013). Caspase-9, caspase-3 and caspase-7 have distinct roles during intrinsic apoptosis. *BMC Cell Biology*.

[39] Tripathi M., Singh B. K., Raisuddin S., Kakkar P. (2011). Abrogation of nimesulide induced oxidative stress and mitochondria mediated apoptosis by *Fumaria parviflora* Lam. extract. *Journal of Ethnopharmacology*.

[40] Hata A. N., Engelman J. A., Faber A. C. (2015). The BCL2 family: key mediators of the apoptotic response to targeted anticancer therapeutics. *Cancer Discovery*.

[41] Chen S., Zhang L., Su Y., Zhang X. (2018). Screening potential biomarkers for colorectal cancer based on circular RNA chips. *Oncology Reports*.

[42] Mao J., Yi M., Tao Y., Huang Y., Chen M. (2019). Costunolide isolated from Vladimiria souliei inhibits the proliferation and induces the apoptosis of HepG2 cells. *Molecular Medicine Reports*.

[43] Mao J., Yi M., Wang R., Huang Y., Chen M. (2018). Protective effects of costunolide against D-galactosamine and lipopolysaccharide-induced acute liver injury in mice. *Frontiers in Pharmacology*.

[44] Nazir S., Nazir S., Kamil S. A. (2014). Pathology and colonization of internal organs after experimental infection of broiler chickens with *Salmonella* gallinarum through oral or intraperitoneal routes. *Revue D’élevage Et De Médecine Vétérinaire Des Pays Tropicaux*.

[45] Withanage G., Wigley P., Kaiser P. (2005). Cytokine and chemokine responses associated with clearance of a primary salmonella enterica Serovar typhimurium infection in the chicken and in protective immunity to rechallenge. *Infection and Immunity*.

[46] Johny A. K., Darre M. J., Hoagland T. A. (2008). Antibacterial effect of trans-cinnamaldehyde on Salmonella enteritidis and campylobacter jejuni in chicken drinking water. *Journal of Applied Poultry Research*.

[47] Sala M., Sunyer J., Herrero C., To-Figueras J., Grimalt J. (2001). Association between serum concentrations of hexachlorobenzene and polychlorobiphenyls with thyroid hormone and liver enzymes in a sample of the general population. *Occupational and Environmental Medicine*.

[48] Johnston D. (1999). Special considerations in interpreting liver function tests. *American Family Physician*.

[49] Wang R., Li S., Jia H. (2021). Protective effects of cinnamaldehyde on the inflammatory response, oxidative stress, and apoptosis in liver of salmonella typhimurium-challenged mice. *Molecules*.

[50] Ruan H., Zhang Z., Tian L., Wang S., Hu S., Qiao J. J. (2016). The *Salmonella* effector SopB prevents ROS-induced apoptosis of epithelial cells by retarding TRAF6 recruitment to mitochondria. *Biochemical and Biophysical Research Communications*.

[51] Waldeck W., Heidenreich E., Mueller G., Wiessler M., Tóth K., Braun K. (2012). ROS-mediated killing efficiency with visible light of bacteria carrying different red fluorochrome proteins. *Journal of Photochemistry & Photobiology B:Biology*.

[52] Yang D., Liang X. C., Shi Y. (2016). Anti-oxidative and anti-inflammatory effects of cinnamaldehyde on protecting high glucose-induced damage in cultured dorsal root ganglion neurons of rats. *Chinese Journal Of Integrative Medicine*.

[53] Wu S., Li Y., Xu Y. (2010). A Salmonella enterica serovar Typhi plasmid induces rapid and massive apoptosis in infected macrophages. *Cellular & Molecular Immunology*.

[54] Lv C., Yuan X., Zeng H. W., Liu R. H., Zhang W. D. (2017). Protective effect of cinnamaldehyde against glutamate-induced oxidative stress and apoptosis in PC12 cells. *European Journal of Pharmacology*.

[55] Lin H. H., Chen H. L., Weng C. C., Janapatla R. P., Chen C. L., Chiu C. H. (2021). Activation of apoptosis by Salmonella pathogenicity island-1 effectors through both intrinsic and extrinsic pathways in Salmonella-infected macrophages. *Journal of Microbiology, Immunology and Infection*.

[56] Grant A. J., Sheppard M., Deardon R. (2008). Caspase-3-dependent phagocyte death during systemic *Salmonella enterica* serovar *Typhimurium* infection of mice. *Immunology*.

[57] Bratburd J. R., Keller C., Vivas E. (2018). Gut Microbial and Metabolic Responses to *Salmonella enterica Serovar Typhimurium* and *Candida albicans*. *MBio*.

[58] Zhang W., Gao J., Shen F. (2020). Cinnamaldehyde changes the dynamic balance of glucose metabolism by targeting ENO1. *Life Sciences*.

[59] Ojcius D. M., Degani H., Mispelter J., Dautry-Varsat A. (1998). Enhancement of ATP levels and glucose metabolism during an infection by chlamydia NMR studies of living cells. *Journal of Biological Chemistry*.

[60] Mathis D., Shoelson S. E. (2011). Immunometabolism: an emerging frontier. *Nature Reviews Immunology*.

[61] Kogut M. H., Genovese K. J., He H., Arsenault R. J. (2016). AMPK and mTOR: sensors and regulators of immunometabolic changes during *Salmonella* infection in the chicken. *Poultry Science*.

[62] Ravindran V., Abdollahi M. R. (2021). Nutrition and digestive physiology of the broiler chick: state of the art and outlook. *Animals*.

